# Supplementary material for: Patterns of genomic deletions in monkeypox virus during the 2022 outbreak in the United States
Source: Nat Commun. 2025 Oct 8;16:8942. doi: 10.1038/s41467-025-64003-y (PMC12508240; doi:10.1038/s41467-025-64003-y)
Supplement: Supplementary file 1 — Supplementary Information [file 41467_2025_64003_MOESM1_ESM.pdf]

Supplemental Material

Data S1. Sample details and additional predicted functional details for MPXV deletions larger than 500 bp.

Data S2. Sequencing details for MPXV sequences with deletions larger than 500 bp. Number of reads corresponding to orthopoxvirus genus using a custom Kraken2 database (from PolkaPox) are included under opxv_reads. The average read depth reported by bwa-mem are listed under avg_depth_bwa. The number of positions with at least 20 reads is reported under count_20xdepth_bwa. MPX Ct value corresponds to average Ct value using CDC-developed clade II-specific MPXV PCR assay for triplicate test. RP: RNase P internal positive control Ct value.

Data S3. Sample details for MPXV sequences used in Figure 3.

Data S4. Sample details for MPXV sequences not generated in this study that were used in Figures 4 and S4.

Data S5. Alignment file for Figure 3

Data S6. Alignment files for Figure 4

Table S1. Primers used to confirm 3,370 and 915 bp deletions and expected product sizes.

Name	Sequence(5′to3′)	PredictedSize:Deletion	Predictedsize:wt
913F_nodeletion	ATGATTGACGGCATCTCCCC		
913R_nodeletion	TGCACTCGAGGAATTATGGCT	N/A	375bp
913F_deletion	TCTCATTCAATTCTTGATTGCAGAG		
913R_deletion	TGACAGATGATCCTGTTCCTG	362bp	1275bp
3370F_deletion	TGCTCAATGGATCATCGTGT		
3370R_deletion	GTAACAAAAGTATTGGTAATTGTGTCA	370bp	3740bp
3370F_nodeletion	TTTCGTCACTTCCACACGCT		
3370R_nodeletion	TCCGCATGGACATCTCCTTT	N/A	361 bp

Table S2. Accession information for NCBI databases.

Accession	Sample Name	Organism	Isolate	BioProject
SAMN49719325	AZ0010	Monkeypox virus	MPXV_USA_2022_AZ0010	PRJNA849962
SAMN49719326	AZ0011	Monkeypox virus	MPXV_USA_2022_AZ0011	PRJNA849962
SAMN49719327	AZ0012	Monkeypox virus	MPXV_USA_2022_AZ0012	PRJNA849962
SAMN49719328	AZ0013	Monkeypox virus	MPXV_USA_2022_AZ0013	PRJNA849962
SAMN49719329	CA0062	Monkeypox virus	MPXV_USA_2022_CA0062	PRJNA849962

SAMN49719330	CA0063	Monkeypox virus	MPXV_USA_2022_CA0063	PRJNA849962
SAMN49719331	CA0064	Monkeypox virus	MPXV_USA_2022_CA0064	PRJNA849962
SAMN49719332	CA0065	Monkeypox virus	MPXV_USA_2022_CA0065	PRJNA849962
SAMN49719333	CA0066	Monkeypox virus	MPXV_USA_2022_CA0066	PRJNA849962
SAMN49719334	CA0067	Monkeypox virus	MPXV_USA_2022_CA0067	PRJNA849962
SAMN49719335	CA0068	Monkeypox virus	MPXV_USA_2022_CA0068	PRJNA849962
SAMN49719336	FL0030	Monkeypox virus	MPXV_USA_2022_FL0030	PRJNA849962
SAMN49719337	GA0027	Monkeypox virus	MPXV_USA_2022_GA0027	PRJNA849962
SAMN49719338	GA0028	Monkeypox virus	MPXV_USA_2022_GA0028	PRJNA849962
SAMN49719339	KY0002	Monkeypox virus	MPXV_USA_2022_KY0002	PRJNA849962
SAMN49719340	LA0005	Monkeypox virus	MPXV_USA_2022_LA0005	PRJNA849962
SAMN49719341	LA0011	Monkeypox virus	MPXV_USA_2022_LA0011	PRJNA849962
SAMN49719342	LA0019	Monkeypox virus	MPXV_USA_2022_LA0019	PRJNA849962
SAMN49719343	LA0020	Monkeypox virus	MPXV_USA_2022_LA0020	PRJNA849962
SAMN49719344	LA0021	Monkeypox virus	MPXV_USA_2022_LA0021	PRJNA849962
SAMN49719345	LA0022	Monkeypox virus	MPXV_USA_2022_LA0022	PRJNA849962
SAMN49719346	LA0023	Monkeypox virus	MPXV_USA_2022_LA0023	PRJNA849962
SAMN49719347	LA0024	Monkeypox virus	MPXV_USA_2022_LA0024	PRJNA849962
SAMN49719348	LA0025	Monkeypox virus	MPXV_USA_2022_LA0025	PRJNA849962
SAMN49719349	LA0026	Monkeypox virus	MPXV_USA_2022_LA0026	PRJNA849962
SAMN49719350	LA0027	Monkeypox virus	MPXV_USA_2022_LA0027	PRJNA849962
SAMN49719351	LA0028	Monkeypox virus	MPXV_USA_2022_LA0028	PRJNA849962
SAMN49719352	LA0029	Monkeypox virus	MPXV_USA_2022_LA0029	PRJNA849962
SAMN49719353	LA0030	Monkeypox virus	MPXV_USA_2022_LA0030	PRJNA849962
SAMN49719354	LA0031	Monkeypox virus	MPXV_USA_2022_LA0031	PRJNA849962
SAMN49719355	LA0032	Monkeypox virus	MPXV_USA_2022_LA0032	PRJNA849962
SAMN49719356	LA0033	Monkeypox virus	MPXV_USA_2022_LA0033	PRJNA849962
SAMN49719357	LA0034	Monkeypox virus	MPXV_USA_2022_LA0034	PRJNA849962
SAMN49719358	LA0035	Monkeypox virus	MPXV_USA_2022_LA0035	PRJNA849962
SAMN49719359	LA0036	Monkeypox virus	MPXV_USA_2022_LA0036	PRJNA849962
SAMN49719360	LA0037	Monkeypox virus	MPXV_USA_2022_LA0037	PRJNA849962
SAMN49719361	LA0038	Monkeypox virus	MPXV_USA_2022_LA0038	PRJNA849962
SAMN49719362	LA0039	Monkeypox virus	MPXV_USA_2022_LA0039	PRJNA849962
SAMN49719363	LA0040	Monkeypox virus	MPXV_USA_2022_LA0040	PRJNA849962
SAMN49719364	LA0043	Monkeypox virus	MPXV_USA_2022_LA0043	PRJNA849962
SAMN49719365	LA0044	Monkeypox virus	MPXV_USA_2022_LA0044	PRJNA849962
SAMN49719366	LA0045	Monkeypox virus	MPXV_USA_2022_LA0045	PRJNA849962
SAMN49719367	MA0004	Monkeypox virus	MPXV_USA_2022_MA0004	PRJNA849962
SAMN49719368	MA0005	Monkeypox virus	MPXV_USA_2022_MA0005	PRJNA849962
SAMN49719369	MA0006	Monkeypox virus	MPXV_USA_2022_MA0006	PRJNA849962
SAMN49719370	MI0015	Monkeypox virus	MPXV_USA_2022_MI0015	PRJNA849962
SAMN49719371	MI0016	Monkeypox virus	MPXV_USA_2022_MI0016	PRJNA849962
SAMN49719372	NC0020	Monkeypox virus	MPXV_USA_2022_NC0020	PRJNA849962

SAMN49719373	NC0021	Monkeypox virus	MPXV_USA_2022_NC0021	PRJNA849962
SAMN49719374	NC0022	Monkeypox virus	MPXV_USA_2022_NC0022	PRJNA849962
SAMN49719375	NJ0019	Monkeypox virus	MPXV_USA_2022_NJ0019	PRJNA849962
SAMN49719376	NM0005	Monkeypox virus	MPXV_USA_2022_NM0005	PRJNA849962
SAMN49719377	NM0006	Monkeypox virus	MPXV_USA_2022_NM0006	PRJNA849962
SAMN49719378	NV0008	Monkeypox virus	MPXV_USA_2022_NV0008	PRJNA849962
SAMN49719379	NV0009	Monkeypox virus	MPXV_USA_2022_NV0009	PRJNA849962
SAMN49719380	NY0056	Monkeypox virus	MPXV_USA_2022_NY0056	PRJNA849962
SAMN49719381	NY0057	Monkeypox virus	MPXV_USA_2022_NY0057	PRJNA849962
SAMN49719382	OH0014	Monkeypox virus	MPXV_USA_2022_OH0014	PRJNA849962
SAMN49719383	OR0010	Monkeypox virus	MPXV_USA_2022_OH0015	PRJNA849962
SAMN49719384	PA0019	Monkeypox virus	MPXV_USA_2022_PA0019	PRJNA849962
SAMN49719385	PA0020	Monkeypox virus	MPXV_USA_2022_PA0020	PRJNA849962
SAMN49719386	TX0068	Monkeypox virus	MPXV_USA_2022_TX0068	PRJNA849962
SAMN49719387	TX0240	Monkeypox virus	MPXV_USA_2022_TX0240	PRJNA849962
SAMN49719388	TX0241	Monkeypox virus	MPXV_USA_2022_TX0241	PRJNA849962
SAMN49719389	TX0242	Monkeypox virus	MPXV_USA_2022_TX0242	PRJNA849962
SAMN49719390	TX0244	Monkeypox virus	MPXV_USA_2022_TX0244	PRJNA849962
SAMN49719391	TX0245	Monkeypox virus	MPXV_USA_2022_TX0245	PRJNA849962
SAMN49719392	VA0013	Monkeypox virus	MPXV_USA_2022_VA0013	PRJNA849962
SAMN49719393	VA0014	Monkeypox virus	MPXV_USA_2022_VA0014	PRJNA849962

Supplementary Methods. Guide for identification and confirmation of large genomic deletions in MPXV.

Figure S1. **Read mapping showing region of poorly mapped reads**. Soft clipped sequence (shown in gray) do not correspond to the reference sequence. Areas where reads only partially map to the reference indicate a genomic change in the sequenced sample relative to the reference being used, so generating a reference-based consensus sequence is not supported by the sequence data. This example read mapping was generated by mapping reads produced from a clade Ia MPXV sample to clade IIb genome MT903344.1. Positions at the top are relative to MT903344.1.

Figure S2. **Visualization of read mapping profile for MPXV sequence data from samples with low overall coverage (average read depth less than 25)**. A. Read mapping profile as displayed in Geneious prime software at the deletion regions for NY0493 (a and b), CA0064 (c), and NM0005 (d). Reads are all soft clippled or masked (pink) at the points where read depth (blue graph) drops to zero, indicating the reads no longer correspond to the reference sequence at that point. Each deletion is supported by >10 reads that span the deletion.

Sequence identity of individual reads to reference MT903344 is shown by shading, where gray indicates identity and black indicates sequence difference.

Figure S3. **Comparison of predicted protein products produced by 3,370 bp and 913 bp deletions compared to homologous products from MPXV and other orthopoxviruses**. a. Translated product of open reading frame produced by 3,370 bp deletion (3370del_predicted_product) corresponds to N-terminus of A51R and C-terminus of A55R homologs in MPXV clade IIb and cowpox virus (CPXV). Amino acid identity to the peptide produced by the 3,370 bp deletion is shown in gray; differences are shown in color. Position of the alignment is shown at the bottom; position in 3370del_predicted_product is shown above. MPXV clade IIb amino acid sequences of ORF at A51R and A55R (partial) based on genome sequence of ON563414; CPXV references AGY97302 (A51R) and UPV00427.1 (A55R) are shown. b. Amino acid alignment shown in A shown wrapped along five lines, with identities to 3370del_predicted_product in gray and differences in black. c. Nucleotide alignment showing the region of the 913 bp deletion compared to several orthpoviruses. Nucleotide identity to the alignment consensus is shown in gray; differences are shown in black; deletions are shown by pink lines. Predicted open reading frames or annotated coding sequences are shown by yellow arrows below gray bars. VARV: Variola virus; TATV: Taterapox virus; RPXV: Rabbitpox virus: BPXV: Buffalopox virus; VACV: Vaccinia virus.

Figure S4. **Complete phylogeny from Figure 4 with accession numbers**.

Figure S5. **Gel image of PCR confirmation of 913 bp (a) and 3,370 bp (b) deletion mutants in ten samples**. Results are shown in the upper gel for the deletion-spanning primer set and in the lower gel for the intra-deletion primer set using primers listed in Table S5. Size of key bands from 1 kb Plus DNA Ladder for Safe Stains (New England Biolabs N05595, L) are noted in bp. Samples are identified by isolate name corresponding to names in Table S1. One sample containing the 913bp deletion showed evidence of amplification of the intra-deletion primer set, suggesting presence of the non-deletion/wildtype (wt) allele for TX0241.

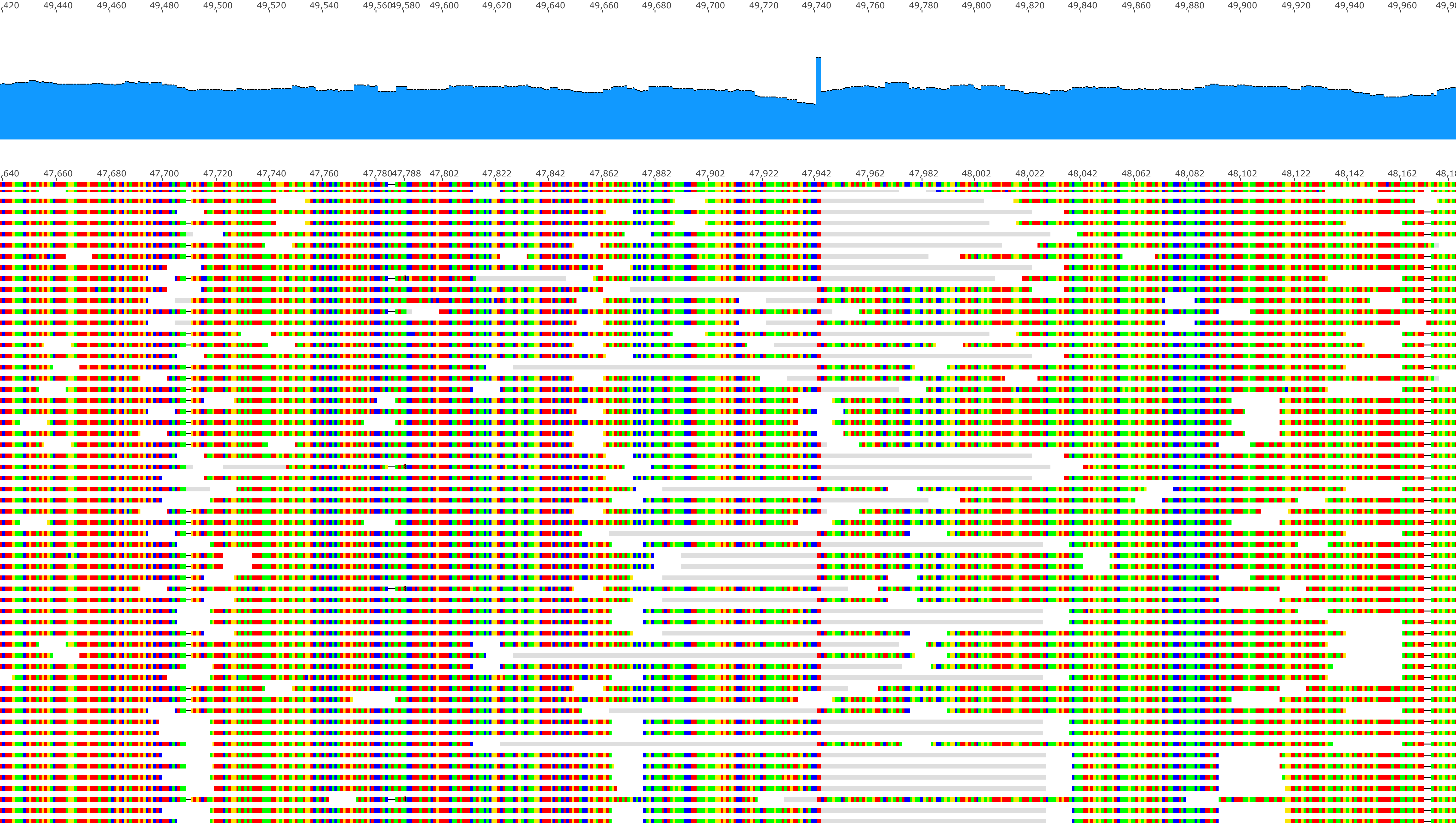
,420 49,440 49,460 49,480 49,500 49,520 49,540 49,560 49,580 49,600 49,620 49,640 49,660 49,680 49,700 49,720 49,740 49,760 49,780 49,800 49,820 49,840 49,860 49,880 49,900 49,920 49,940 49,960
,640 47,660 47,680 47,700 47,720 47,740 47,760 47,780 47,788 47,802 47,822 47,842 47,862 47,882 47,902 47,922 47,942 47,962 47,982 48,002 48,022 48,042 48,062 48,082 48,102 48,122 48,142 48,162

Figure S1. **Read mapping showing region of poorly mapped reads**. Soft clipped sequence (shown in gray) do not correspond to the reference sequence. Areas where reads only partially map to the reference indicate a genomic change in the sequenced sample relative to the reference being used, so generating a reference-based consensus sequence is not supported by the sequence data. This example read mapping was generated by mapping reads produced from a clade Ia MPXV sample to clade IIb genome MT903344.1. Positions at the top are relative to MT903344.1.

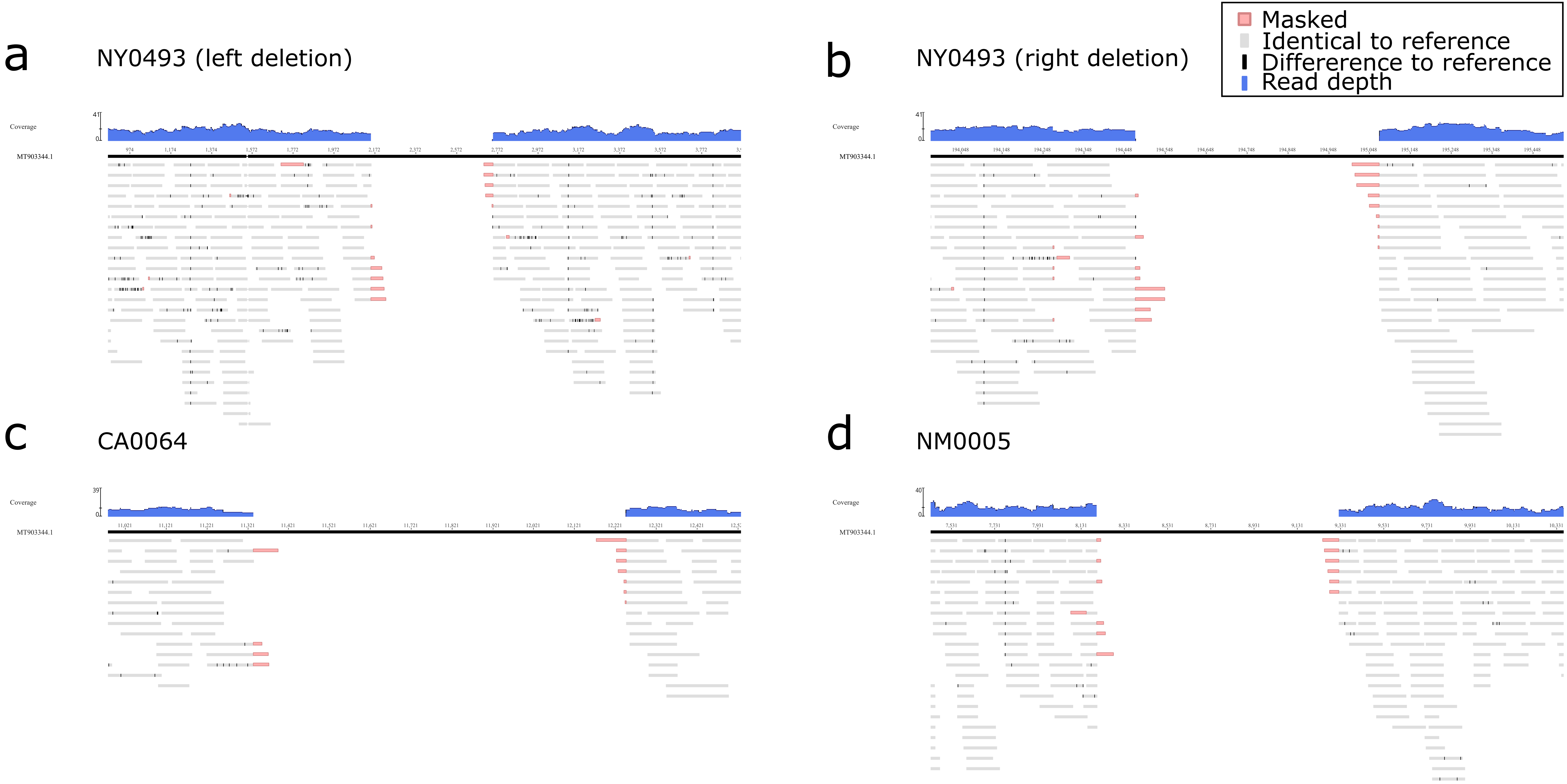
Masked
Identical to reference
Differerence to reference
Read depth
a
NY0493 (left deletion)
b
NY0493 (right deletion)
c
CA0064
d
NM0005
Coverage
MT903344.1

Figure S2. **Visualization of read mapping profile for MPXV sequence data from samples with low overall coverage (average read depth less than 25)**. A. Read mapping profile as displayed in Geneious prime software at the deletion regions for NY0493 (a and b), CA0064 (c), and NM0005 (d). Reads are all soft clippled or masked (pink) at the points where read depth (blue graph) drops to zero, indicating the reads no longer correspond to the reference sequence at that point. Each deletion is supported by >10 reads that span the deletion. Sequence identity of individual reads to reference MT903344 is shown by shading, where gray indicates identity and black indicates sequence difference.

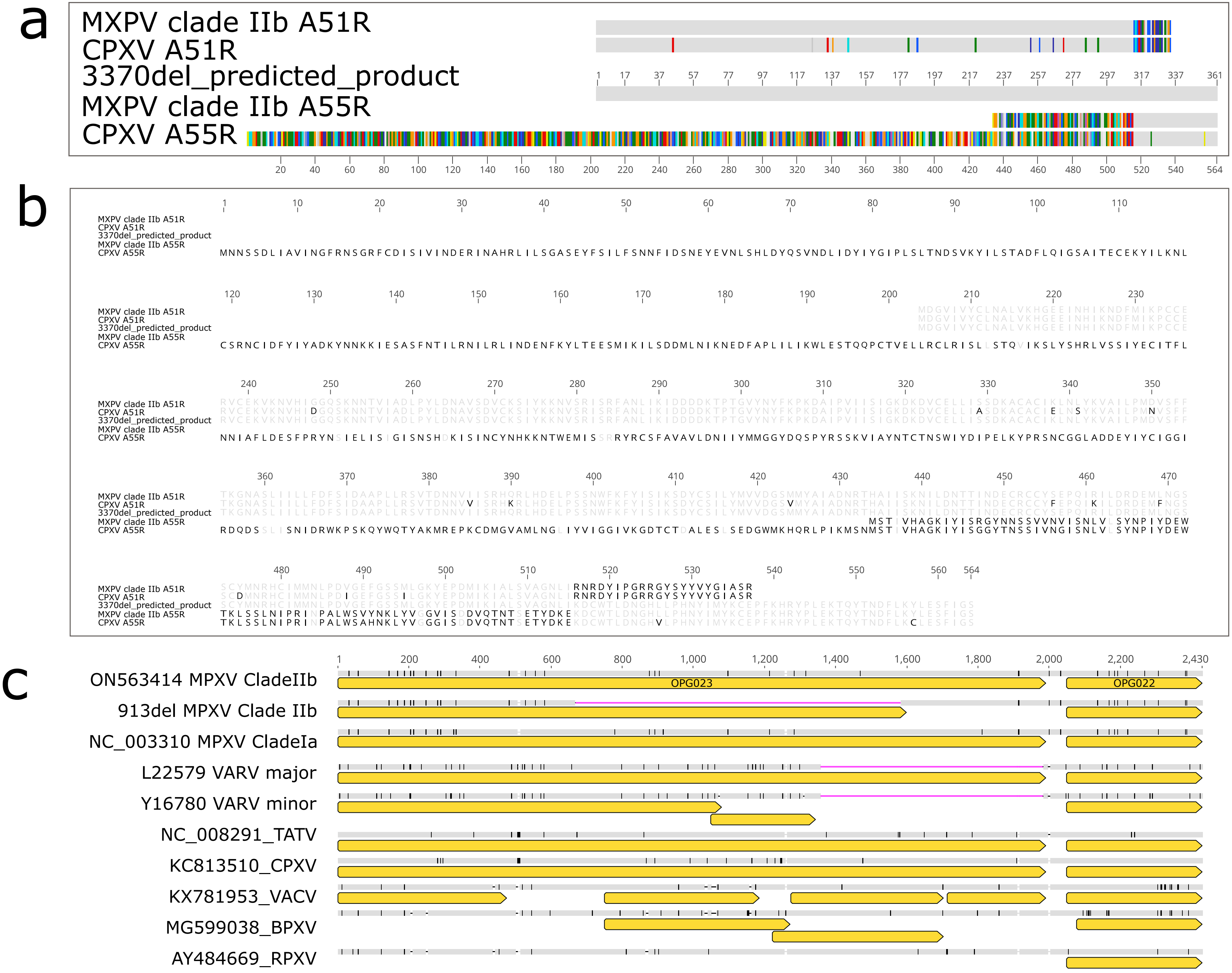
a
MXPV clade IIb A51R
CPXV A51R
3370del_predicted_product
MXPV clade IIb A55R
CPXV A55R
b
MXPV clade IIb A51R
CPXV A51R
3370del_predicted_product
MXPV clade IIb A55R
CPXV A55R
c
ON563414 MPXV CladeIIb
913del MPXV Clade IIb
NC_003310 MPXV CladeIa
L22579 VARV major
Y16780 VARV minor
NC_008291_TATV
KC813510_CPXV
KX781953_VACV
MG599038_BPXV
AY484669_RPXV
OPG023
OPG022

Figure S3. **Comparison of predicted protein products produced by 3,370 bp and 913 bp deletions compared to homologous products from MPXV and other orthopoxviruses**. a. Translated product of open reading frame produced by 3,370 bp deletion (3370del_predicted_product) corresponds to N-terminus of A51R and C-terminus of A55R homologs in MPXV clade IIb and cowpox virus (CPXV). Amino acid identity to the peptide produced by the 3,370 bp deletion is shown in gray; differences are shown in color. Position of the alignment is shown at the bottom; position in 3370del_predicted_product is shown above. MPXV clade IIb amino acid sequences of ORF at A51R and A55R (partial) based on genome sequence of ON563414; CPXV references AGY97302 (A51R) and UPV00427.1 (A55R) are shown. b. Amino acid alignment shown in A shown wrapped along five lines, with identities to 3370del_predicted_product in gray and differences in black. c. Nucleotide alignment showing the region of the 913 bp deletion compared to several orthpoviruses. Nucleotide identity to the alignment consensus is shown in gray; differences are shown in black; deletions are shown by pink lines. Predicted open reading frames or annotated coding sequences are shown by yellow arrows below gray bars. VARV: Variola virus; TATV: Taterapox virus; RPXV: Rabbitpox virus: BPXV: Buffalopox virus; VACV: Vaccinia virus.

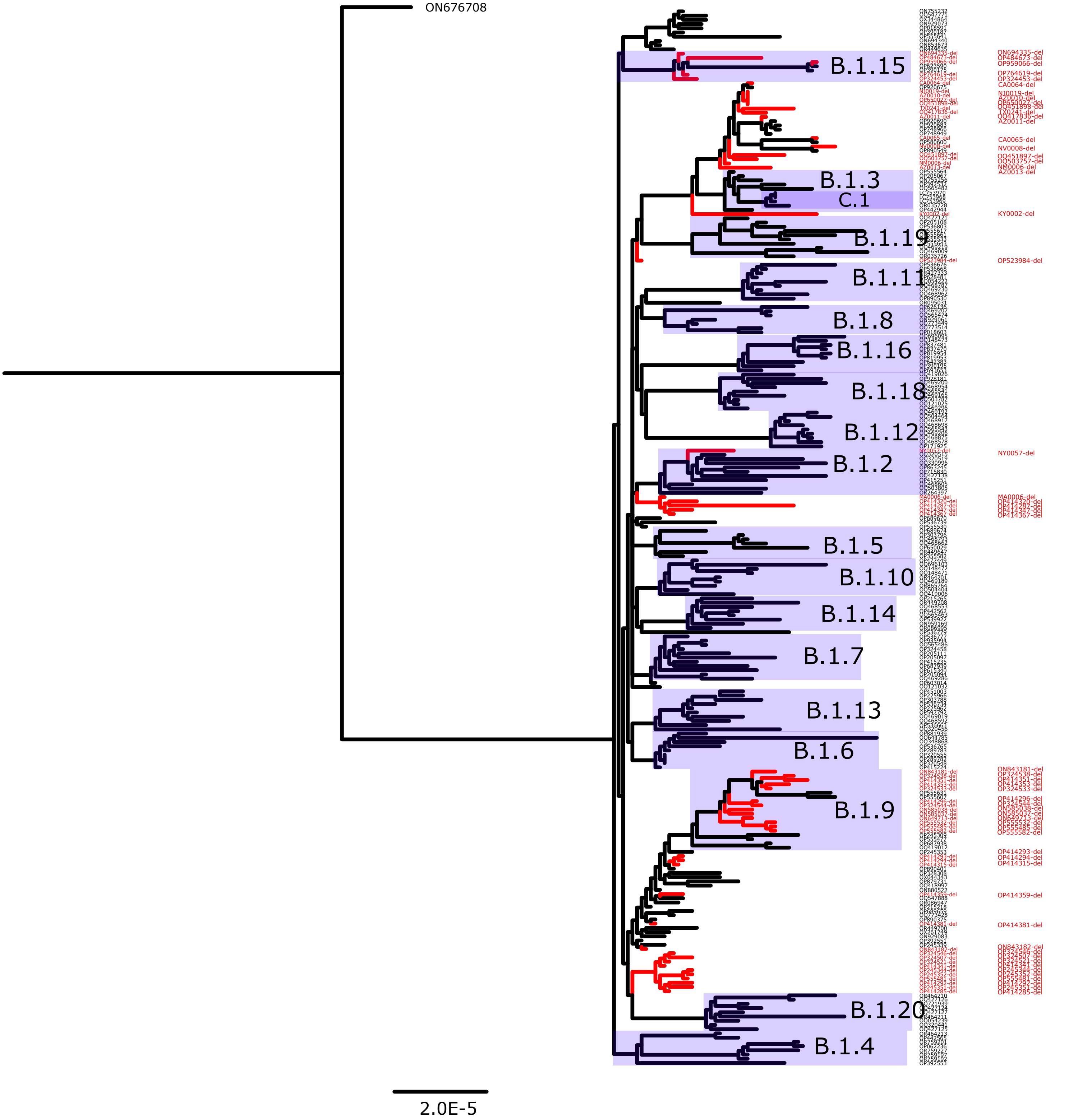
ON676708
B.1.15
B.1.3
C.1
B.1.19
B.1.11
B.1.8
B.1.16
B.1.18
B.1.12
B.1.2
B.1.5
B.1.10
B.1.14
B.1.7
B.1.13
B.1.6
B.1.9
B.1.20
B.1.4
2.0E-5
ON694335-del
OP484673-del
OP959066-del
OP764619-del
OP324453-del
CA0064-del
NJ0019-del
AZ0010-del
OP650022-del
OQ451898-del
TX0241-del
OQ417836-del
AZ0011-del
CA0065-del
NV0008-del
OQ451897-del
OQ503757-del
NM0006-del
AZ0013-del
KY0002-del
OP523984-del
NY0057-del
MA0006-del
OP414320-del
OP414287-del
OP414347-del
OP414367-del
ON843181-del
OP324538-del
OP414351-del
OP414353-del
OP324533-del
OP414296-del
OP324544-del
ON585038-del
ON585037-del
ON649713-del
OP555532-del
OP555485-del
OP555582-del
OP414293-del
OP414294-del
OP414315-del
OP414359-del
OP414381-del
ON843182-del
OP324546-del
OP324507-del
OP324521-del
OP414341-del
OP245344-del
OP555481-del
OP414292-del
OP245351-del
OP414285-del

Figure S4. **Complete phylogeny from Figure 4 with accession numbers**.

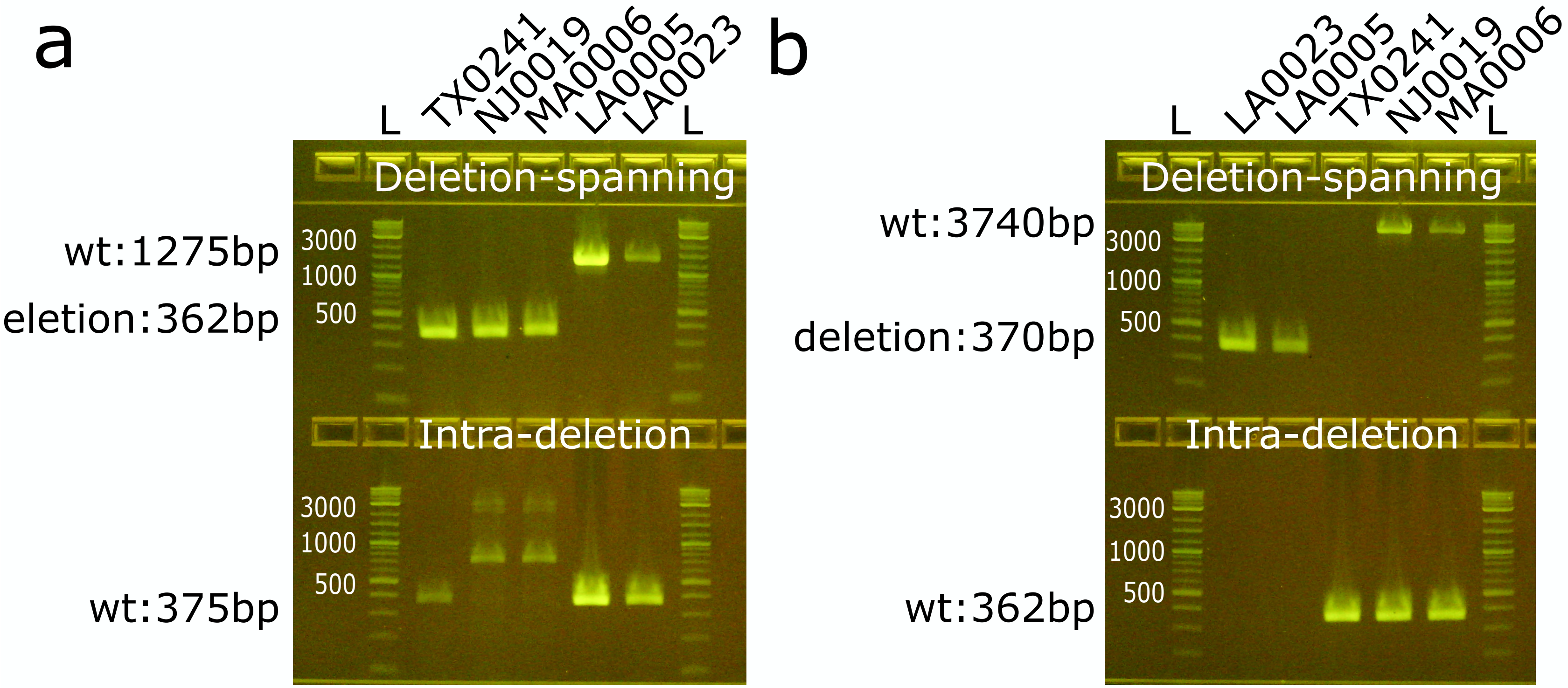
a
L
TX0241
NJ0019
MA0006
LA0005
LA0023
L
Deletion-spanning
wt:1275bp
eletion:362bp
3000
1000
500
Intra-deletion
3000
1000
500
wt:375bp
b
L
LA0023
LA0005
TX0241
NJ0019
MA0006
L
Deletion-spanning
wt:3740bp
3000
1000
500
deletion:370bp
Intra-deletion
3000
1000
500
wt:362bp

Figure S5. **Gel image of PCR confirmation of 913 bp (a) and 3,370 bp (b) deletion mutants in ten samples**. Results are shown in the upper gel for the deletion-spanning primer set and in the lower gel for the intra-deletion primer set using primers listed in Table S5. Size of key bands from 1 kb Plus DNA Ladder for Safe Stains (New England Biolabs N05595, L) are noted in bp. Samples are identified by isolate name corresponding to names in Table S1. One sample containing the 913bp deletion showed evidence of amplification of the intra-deletion primer set, suggesting presence of the non-deletion/wildtype (wt) allele for TX0241.

Supplementary Methods: Guide for identification and confirmation of large genomic deletions in MPXV.

To identify large deletions in the MPXV genome among U.S. mpox samples, read mapping profiles from all samples from successful MPXV genomic sequencing runs were examined by eye. Reads mapped to clade II reference genome or MT903344 using bwa mem were visualized using CLC Genomics Workbench 23 or 24 (Qiagen) or Geneious Prime (GraphPad Sorftware LLC). Potential deletions were identified as dips in read depth over small portions of the genome. These suspect deletion regions are easier to visualize in samples with higher average read depth. Ability to distinguish and confirm deletions in samples with average read depth <35 was challenging, so samples were required to have at least 10 reads spanning the deletion to be included as deletions. One exception to this was LA0044, which only had three reads supporting the deletion and an overall average read depth of 6. This sample was included as an exception for the purpose of surveillance of a previously established deletion in the state of Louisiana. In some cases, samples with low overall read depth were re-sequenced to achieve higher read depth as part of normal sequence surveillance efforts; however, it is possible additional deletions were missed.

Next, areas of low read depth were investigated to determine if the lapse in coverage was due to lack of reads or a true deletion. Low coverage would be indicated by lower number of reads, while deletions can be identified by reads that only partially map to the genome at the left and right borders of the suspected deletion. In Supplemental Figure S2 and the figure below, this is shown by the pink underlined portions of the reads. All reads stop matching the reference sequence at the same exact position (after GTC). Then, all reads have the same sequence. If we search for that sequence (starting with gray text CAAAC), we see that it corresponds to the position in the reference at the other side of the deletion.

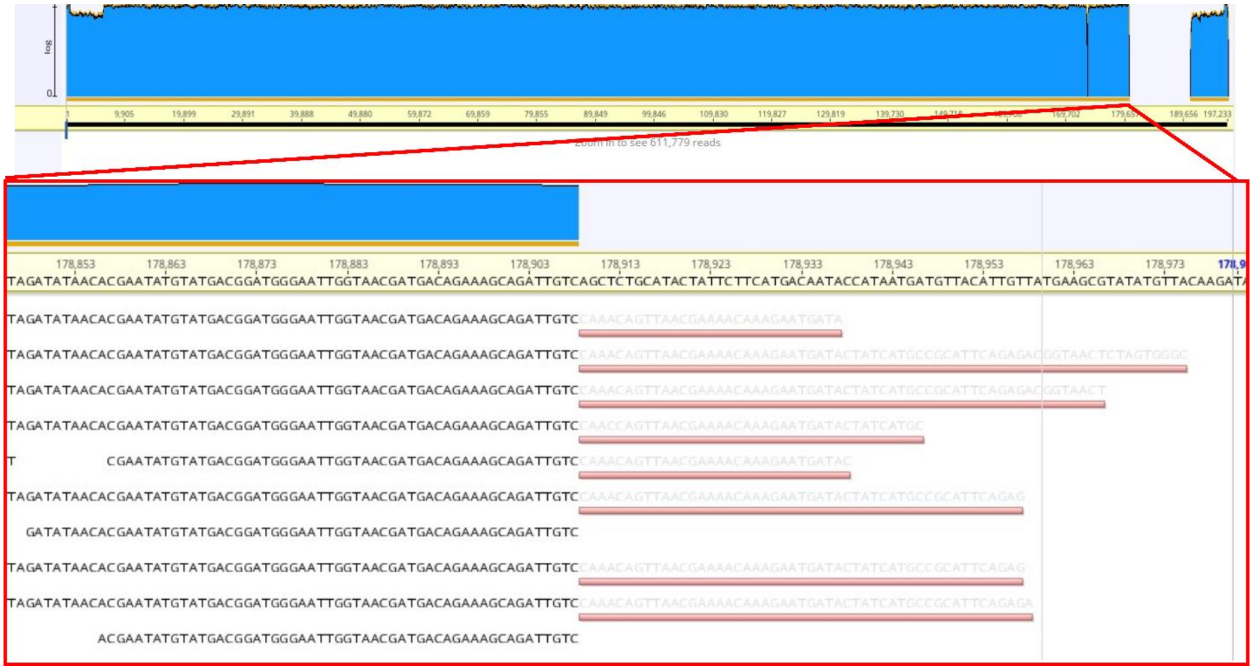

Figure. Visualization of read mapping profile for MPXV sequence data with a suspected large genomic deletion. A. Whole genome read mapping profile. B. Zoomed in visualization of the left side of the deletion, showing all reads at the deletion interface are clipped or masked (shown by pink bar and gray text). Read sequence corresponding to the reference sequence is shown in black text. Reference sequence is highlighted in the yellow box.

Next, a draft genome containing the proposed deletion is generated, either from PolkaPox (http://github.com/CDCgov/polkapox) or through manual assembly of unicycler contigs output from PolkaPox. Draft genomes were examined to confirm the deletion junction sequence by searching the draft genome for the sequence from the read mapping profile. In the figure above, this sequence would include: CAGATTGTCCAAACAGTT. Orthopoxvirus reads were then mapped to that draft genome using bwa mem (https://github.com/lh3/bwa, bwa v 0.7.16). The read depth coverage profiles were visualized to ensure evenness across the draft genomes. The deletion junction was examined to make sure the reads mapped across the deletion evenly, with no partial mapping of reads. A minimum of 10 reads spanning the deletion junction and >50% of reads at the junction supporting the deletion were required to confirm presence of the deletion.

Rarely, reads did not support the existence of a simple deletion, and those samples were excluded from this study. An example is shown in Supplemental Figure S1, showing an example of an incorrect junction where no reads map across. Reads that correspond to the draft genome are shown in colored boxes with clipped portions of reads that did not align shown in gray. In the figure, reads that corresponded to the draft genome to the left of the junction point at approximate position 47,943 did not correspond to the draft genome sequence to the right of the red arrow. The same is true for reads from the other side, suggesting the draft genome is incorrect and must be fixed. This signal is indicative of a more complex rearrangement or incorrect junction points of the deletion. Again, any sample with this signal was not included in the final tally of deletion genomes and was removed from the study.

A subset of deletions underwent additional characterization and verification. Three samples underwent long read sequencing using Oxford Nanopore technology. Library preparation was performed on extracted DNA using Ligation Sequencing kit (Oxford Nanopore Technologies SQK-LSK-109) following the manufacturer's protocol for genomic DNA. Libraries were sequenced (one sample per flow cell) on a MinION sequencer or GridION sequencer using a MIN109 R9.4.1 flow cell (Oxford Nanopore). Basecalling was performed using guppy version 6.1.2 with high accuracy and qscore filtering (for MinION runs) or was performed on GridION using high accuracy basecalling. Reads were mapped to the final genome produced from short read sequencing using minimap2 v 2.16 (https://github.com/lh3/minimap2).

Existence of the 913 and 3,370 bp deletions were confirmed by PCR. Primers were designed for the 3,370 and 913 bp deletion regions using Primer3 v 2.3.5 in Geneious Prime. Primer sequences can be found in Table S1. Five samples containing each deletion were subject to PCR using each of four primer sets designed to amplify the deletion and wildtype sequence at each locus. PCR was performed using NEBNext Ultra II Q5 Master Mix (M0544 New England Biolabs) following the manufacturer's recommendations. 50 µL reactions were prepared using 25 µL Ultra II Q5 Master Mix, 2.5 µL each forward and reverse primer at 10 µM, 18 µL nuclease free water and 2 µL template DNA. PCR was performed by denaturation at 98°C for 30 seconds followed by 35 cycles of 98°C for 10 seconds, 62°C for 30 seconds and 72°C for 30 seconds, followed by 2 minutes final extension at 72°C.
