# Supplementary material for: Patterns of genomic deletions in monkeypox virus during the 2022 outbreak in the United States
Source: Nat Commun. 2025 Oct 8;16:8942. doi: 10.1038/s41467-025-64003-y (PMC12508240; doi:10.1038/s41467-025-64003-y)
Supplement: Supplementary file 8 — Reporting Summary [file 41467_2025_64003_MOESM8_ESM.pdf]

nature portfolio

Corresponding author(s): Crystal Gigante

Last updated by author(s): Aug 27, 2025

Reporting Summary

Nature Portfolio wishes to improve the reproducibility of the work that we publish. This form provides structure for consistency and transparency in reporting. For further information on Nature Portfolio policies, see our Editorial Policies and the Editorial Policy Checklist.

Statistics

For all statistical analyses, confirm that the following items are present in the figure legend, table legend, main text, or Methods section.

n/a	Confirmed	
☐	☒	The exact sample size (*n*) for each experimental group/condition, given as a discrete number and unit of measurement
☐	☒	A statement on whether measurements were taken from distinct samples or whether the same sample was measured repeatedly
☐	☒	The statistical test(s) used AND whether they are one- or two-sided<br>*Only common tests should be described solely by name; describe more complex techniques in the Methods section.*
☐	☒	A description of all covariates tested
☐	☒	A description of any assumptions or corrections, such as tests of normality and adjustment for multiple comparisons
☐	☒	A full description of the statistical parameters including central tendency (e.g. means) or other basic estimates (e.g. regression coefficient) AND variation (e.g. standard deviation) or associated estimates of uncertainty (e.g. confidence intervals)
☐	☒	For null hypothesis testing, the test statistic (e.g. *F*, *t*, *r*) with confidence intervals, effect sizes, degrees of freedom and *P* value noted<br>*Give P values as exact values whenever suitable.*
☒	☐	For Bayesian analysis, information on the choice of priors and Markov chain Monte Carlo settings
☒	☐	For hierarchical and complex designs, identification of the appropriate level for tests and full reporting of outcomes
☒	☐	Estimates of effect sizes (e.g. Cohen's *d*, Pearson's *r*), indicating how they were calculated

Our web collection on statistics for biologists contains articles on many of the points above.

Software and code

Policy information about availability of computer code

Data collection	Details are included in the manuscript
Data analysis	Details are included in the manuscript

For manuscripts utilizing custom algorithms or software that are central to the research but not yet described in published literature, software must be made available to editors and reviewers. We strongly encourage code deposition in a community repository (e.g. GitHub). See the Nature Portfolio guidelines for submitting code & software for further information.

Data

Policy information about availability of data

All manuscripts must include a data availability statement. This statement should provide the following information, where applicable:

- Accession codes, unique identifiers, or web links for publicly available datasets
- A description of any restrictions on data availability
- For clinical datasets or third party data, please ensure that the statement adheres to our policy

Assembled genomes and raw read data generated in this study were submitted deposited to NCBI SRA and Genbank under accessions listed in Text S2. Accession numbers used in phylogenetic analysis are listed in Data S3. Alignments used for phylogenetic analyses are available in at Dryad (Pending ACCESSION)Data S5 and S6. MPXV genome sequences were assembled using PolkaPox v0.1-beta (https://github.com/CDCgov/polkapox). Genome annotation and submission to NCBI databases was performed using TOSTADAS (https://github.com/CDCgov/tostadas).

Research involving human participants, their data, or biological material

Policy information about studies with human participants or human data. See also policy information about sex, gender (identity/presentation), and sexual orientation and race, ethnicity and racism.

Reporting on sex and gender	N/A
Reporting on race, ethnicity, or other socially relevant groupings	N/A
Population characteristics	N/A
Recruitment	N/A
Ethics oversight	N/A

Note that full information on the approval of the study protocol must also be provided in the manuscript.

Field-specific reporting

Please select the one below that is the best fit for your research. If you are not sure, read the appropriate sections before making your selection.

- [x] Life sciences
- [ ] Behavioural & social sciences
- [ ] Ecological, evolutionary & environmental sciences

For a reference copy of the document with all sections, see nature.com/documents/nr-reporting-summary-flat.pdf

Life sciences study design

All studies must disclose on these points even when the disclosure is negative.

Sample size	No sample size calculation was performed. Sample size determined by the total number of samples received for clade-surveillance of MPXV samples at CDC during a specified timeframe (2022 to 2023). All samples that met the qPCR value threshold for sequencing in our laboratory were included. The sample size is the largest ever analyzed in this way.
Data exclusions	MPXV samples exhibiting large genomic rearrangements were excluded from this analysis as the data have revealed a case correlate contributer in those cases we are still investigating.
Replication	10% of genomic deletions were confirmed through independent sequencing by a different technology/method, re-sequencing or by PCR.
Randomization	N/A all samples were examined for the presence of large genomic deletions. The study did not contain groups.
Blinding	N/A The study did not contain groups. Researchers were not aware of the deletions prior to analyzing the data.

Reporting for specific materials, systems and methods

We require information from authors about some types of materials, experimental systems and methods used in many studies. Here, indicate whether each material, system or method listed is relevant to your study. If you are not sure if a list item applies to your research, read the appropriate section before selecting a response.

Materials & experimental systems

n/a	Involved in the study	
x		Antibodies
x		Eukaryotic cell lines
x		Palaeontology and archaeology
x		Animals and other organisms
x		Clinical data
x		Dual use research of concern
x		Plants

Methods

n/a	Involved in the study	
x		ChIP-seq
x		Flow cytometry
x		MRI-based neuroimaging

Plants

Seed stocks	N/A
Novel plant genotypes	N/A
Authentication	N/A
