# Supplementary material for: Patterns of genomic deletions in monkeypox virus during the 2022 outbreak in the United States
Source: Nat Commun. 2025 Oct 8;16:8942. doi: 10.1038/s41467-025-64003-y (PMC12508240; doi:10.1038/s41467-025-64003-y)
Supplement: Supplementary file 10 — Description of Additional Supplementary Files [file 41467_2025_64003_MOESM10_ESM.pdf]

Description of Additional Supplementary Files

File Name: Supplementary Data 1
Description: Sample details and additional predicted functional details for MPXV deletions larger than 500 bp.

File Name: Supplementary Data 2
Description: Sequencing details for MPXV sequences with deletions larger than 500 bp. Number of reads corresponding to orthopoxvirus genus using a custom Kraken2 database (from PolkaPox) are included under opxv_reads. The average read depth reported by bwa-mem are listed under avg_depth_bwa. The number of positions with at least 20 reads is reported under count_20xdepth_bwa. MPX Ct value corresponds to average Ct value using CDC-developed clade II-specific MPXV PCR assay for triplicate test. RP: RNase P internal positive control Ct value.

File Name: Supplementary Data 3
Description: Sample details for MPXV sequences used in Figure 3.

File Name: Supplementary Data 4
Description: Sample details for MPXV sequences not generated in this study that were used in Figures 4 and S4

File Name: Supplementary Data 5
Description: Alignment file for Figure 3

File Name: Supplementary Data 6
Description: Alignment files for Figure 4
